# Correction: Retinoic acid-gated BDNF synthesis in neuronal dendrites drives presynaptic homeostatic plasticity

**DOI:** 10.7554/eLife.91539

**Published:** 2023-08-02

**Authors:** Shruti Thapliyal, Kristin L Arendt, Anthony G Lau, Lu Chen

**Keywords:** Mouse

Thapliyal S, Arendt KL, Lau AG, Chen L. 2022. Retinoic acid-gated BDNF synthesis in neuronal dendrites drives presynaptic homeostatic plasticity. *eLife* **11**:e79863. doi: 10.7554/eLife.79863.

Published 14 December 2022

We have recently discovered that several data points from different experiments in Figure 1 and Figure 3 are identical and thus represent duplicate data sets in our electrophysiology experiments. We have investigated this carefully and found several copy-and-paste errors that occurred during data organization and resulted in these duplicates. To help understand how this type of mistake happened, we made a summary of our standard workflow for data collection and analysis of miniature synaptic responses.

1. Cells are recorded using Clampex on PC computers. Files generated in Clampex are opened in Clampfit to be exported into an abf integer format (Clampex generates them in a floating-point format) that is compatible with our mini analysis software by Synaptosoft.
2. Converted abf files are opened in Mini Analysis. Events are selected based on their kinetic properties with a minimum threshold amplitude set in the program.
3. Processed miniature response data are copy and pasted into Excel (Windows XP edition) for further analysis. The average amplitude and frequency of each cell are calculated in Excel. From here the individual cells are transferred into a summary sheet for the particular experiment sets.
4. Once an experimental set is processed in excel, the data is then transferred into Prism (MacOS) to compile preliminary figures and statistical analysis.

Large data sets including multiple independent biological replicates are compiled into prism sheets. Data is then pooled for figure generation, Individual columns per experimental group must be copied into a single column for this purpose.

We use SigmaPlot combined with Adobe Illustrator for generating publication-quality figures. To generate source data files for final figure generation in SigmaPlot, Prism compilations are copied into Excel (MacOS). We use the ‘sort’ function in Excel to efficiently get rid of the empty spreadsheet cells between data points.

Figures made in SigmaPlot are finalized in Adobe Illustrator.

We realize that the data processing steps we used for this type of data – some of which are further complicated since data collection is done on PCs and analyses are done on Mac computers - are not ideal and may have contributed to the copy-and-paste errors in this publication. We take full responsibility for the errors we made. They have all been corrected in the article. The conclusions of the study are unaffected by the correction.

The specific errors and corrections are listed below.

In Figure 1B, three data points in the postsynaptic RARα KO DMSO group (ctrl for RA treatment) were mistakenly copied from the postsynaptic RARα KO DMSO group for Figre 1C (ctrl for CNQX treatment) during data organization. We have taken out the three data sets from this group in the corrected Figure 1B.

The corrected Figure 1 is shown here:

**Figure 1:**
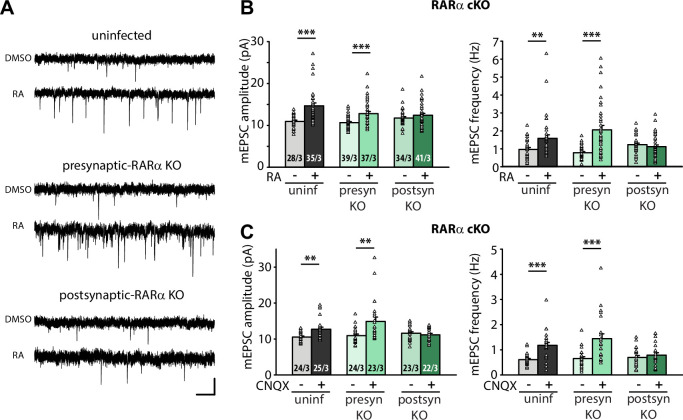


The original incorrect Figure 1 is shown here for reference:

**Figure 2:**
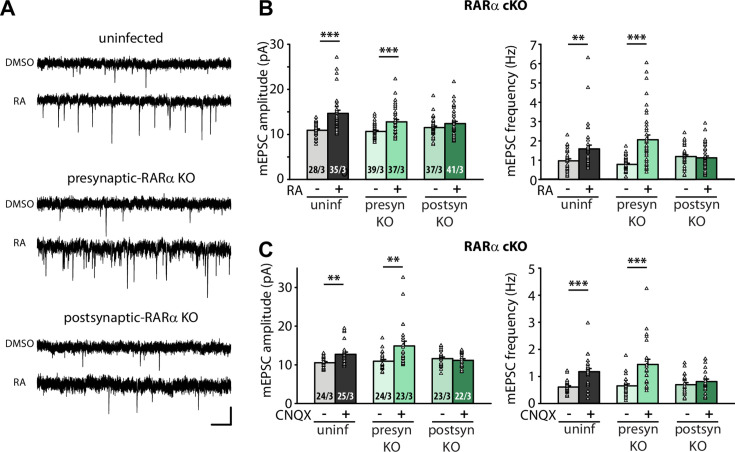


The corrected legend for Figure 1 should now read:

**Figure 1. Postsynaptic RARα expression is required for presynaptic homeostatic plasticity.** (**A**) Example traces of mEPSCs recorded from hippocampal pyramidal neurons in organotypic slices from WT (uninfected), presynaptic RARα KO (Cre expression in CA3) and postsynaptic RARα KO (Cre expression in CA1) groups treated with DMSO or RA (10 μM, 4 hr). Scale bars, 10 pA, 0.5 sec. (**B**) Quantification of mEPSC amplitudes and frequencies recorded from WT, presynaptic and postsynaptic RARα KO neurons treated with DMSO or RA. **, *P*<0.01; ***, *P*<0.001; two-way ANOVA followed by Mann Whitney test. Amp: F(2,208) = 5.413, *P*<0.01; freq: F(2,208) = 11.81, *P*<0.0001. (**C**) Quantification of mEPSC amplitudes and frequencies recorded from WT, presynaptic and postsynaptic RARα KO neurons treated with DMSO or CNQX (36 hours). **, *P*<0.01; ***, *P*<0.001; two-way ANOVA followed by Mann Whitney test. Amp: F(2,135) = 6.004, *P*<0.01; freq: F(2,135) = 5.23, *P*<0.01. n/N represent number of neurons/number of independent experiments (pups). All graphs represent mean ± SEM.

The original legend for Figure 1 is shown for reference:

**Figure 1. Postsynaptic RARα expression is required for presynaptic homeostatic plasticity.** (**A**) Example traces of mEPSCs recorded from hippocampal pyramidal neurons in organotypic slices from WT (uninfected), presynaptic RARα KO (Cre expression in CA3) and postsynaptic RARα KO (Cre expression in CA1) groups treated with DMSO or RA (10 μM, 4 hr). Scale bars, 10 pA, 0.5 sec. (**B**) Quantification of mEPSC amplitudes and frequencies recorded from WT, presynaptic and postsynaptic RARα KO neurons treated with DMSO or RA. **, *P*<0.01; ***, *P*<0.001; two-way ANOVA followed by Tukey’s test. Amp: F(2,211) = 4.79, *P*<0.01; freq: F(2,211) = 11.47, *P*<0.0001. (**C**) Quantification of mEPSC amplitudes and frequencies recorded from WT, presynaptic and postsynaptic RARα KO neurons treated with DMSO or CNQX (36 hours). **, *P*<0.01; ***, *P*<0.001; two-way ANOVA followed by Tukey’s test. Amp: F(2,135) = 6.00, *P*<0.01; freq: F(2,135) = 4.55, *P*=0.01. n/N represent number of neurons/number of independent experiments (pups). All graphs represent mean ± SEM.

There are three mistakes in Figure 3.

In Figure 3C and 3F, of the three independent biological replicates, one set of the mEPSC frequency data was left out and replaced by the wrong set during data exporting and organization from PRISM to EXCEL. The amplitude data were not affected. We have replaced the incorrected dataset with the correct ones.

A second mistake is in Figure 3E and 3F, WT (uninfected) DMSO and treatment groups for both RA and CNQX experiment have mistakenly included a subset of data for the same group in 3 C. This was caused by copying the data from the wrong tab of the EXCEL files as the labels for the columns were identical on these different tabs of sheets (same experimental manipulations in different mouse lines). We have corrected this error by taking out the wrong set and replaced with the correct set that was left out from the original files.

A third mistake is in Figure 3F postsynaptic TrkB KO CNQX treated group, four data points were copy and pasted twice (duplication error) and these four duplicates have now been removed.

The corrected Figure 3 is shown here:

**Figure 3:**
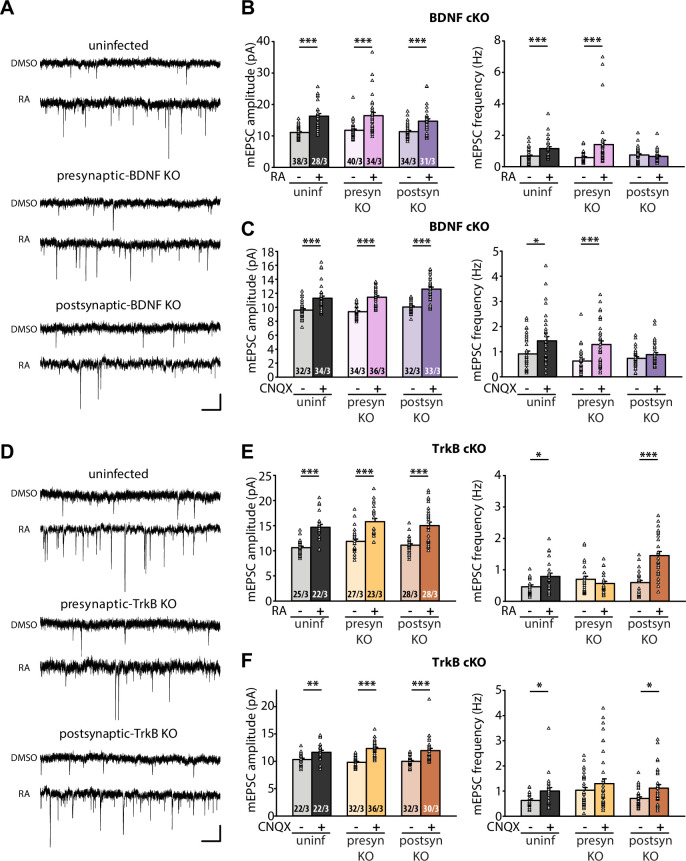


The original incorrect Figure 3 is shown here for reference:

**Figure 4:**
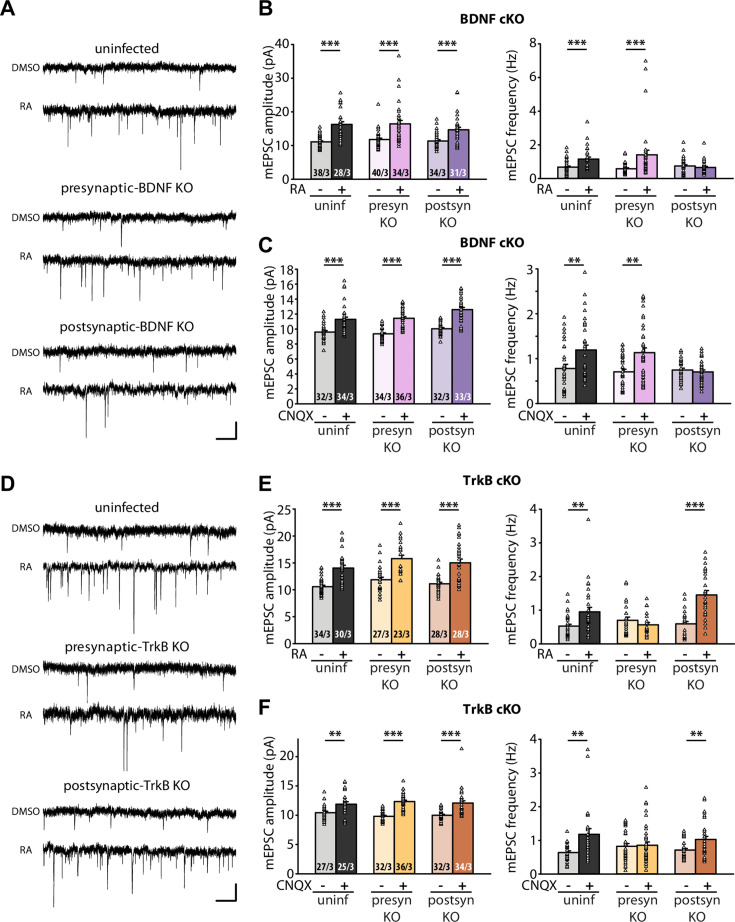


The corrected legend for Figure 3 should now read:

**Figure 3. Retrograde BDNF signalling is required for RARα-mediated regulation of presynaptic homeostatic scaling.** (**A**) Example traces of mEPSCs recorded from hippocampal pyramidal neurons in organotypic slices from WT (uninfected), presynaptic BDNF KO (Cre expression in CA3) and postsynaptic BDNF KO (Cre expression in CA1) groups treated with DMSO or RA (10 μM, 4 hr). Scale bars, 10 pA, 0.5 sec. (**B**) Quantification of mEPSC amplitudes and frequencies recorded from WT, presynaptic and postsynaptic BDNF KO neurons treated with DMSO or RA. ***, *P*<0.001; two-way ANOVA followed by Mann Whitney test. Amp: F(2,199) = 1.062, *P*>0.3; freq: F(2,199) = 6.244, *P*<0.01. (**C**) Quantification of mEPSC amplitudes and frequencies recorded from WT, presynaptic and postsynaptic BDNF KO neurons treated with DMSO or CNQX (36 hours). *, *P*<0.05, **, *P*<0.01; ***, *P*<0.001; two-way ANOVA followed by Mann Whitney test. Amp: F(2,195) = 1.77, *P*>0.15; freq: F(2,195) = 2.53, *P*>0.05. (**D**) Example traces of mEPSCs recorded from hippocampal pyramidal neurons in organotypic slices from WT (uninfected), presynaptic TrkB KO (Cre expression in CA3) and postsynaptic TrkB KO (Cre expression in CA1) groups treated with DMSO or RA (10 μM, 4 hr). Scale bars, 10 pA, 0.5 sec. (**E**) Quantification of mEPSC amplitudes and frequencies recorded from WT, presynaptic and postsynaptic TrkB KO neurons treated with DMSO or RA. **, *P*<0.01; ***, *P*<0.001; two-way ANOVA followed by Mann Whitney test. Amp: F(2,147) = 0.01, *P*>0.9; freq: F(2,147) = 14.87, *P*<0.0001. (**F**) Quantification of mEPSC amplitudes and frequencies recorded from WT, presynaptic and postsynaptic TrkB KO neurons treated with DMSO or CNQX (36 hours). *, *P*<0.05,**, *P*<0.01; ***, *P*<0.001; two-way ANOVA followed by Mann Whitney test. Amp: F(2,168) = 2.33, *P*>0.1; freq: F(2,168) = 0.17, *P*>0.8.

n/N represent number of neurons/number of independent experiments. All graphs represent mean ± SEM.

The original legend for Figure 3 is shown for reference:

**Figure 3. Retrograde BDNF signalling is required for RARα-mediated regulation of presynaptic homeostatic scaling.** (**A**) Example traces of mEPSCs recorded from hippocampal pyramidal neurons in organotypic slices from WT (uninfected), presynaptic BDNF KO (Cre expression in CA3) and postsynaptic BDNF KO (Cre expression in CA1) groups treated with DMSO or RA (10 μM, 4 hr). Scale bars, 10 pA, 0.5 sec. (**B**) Quantification of mEPSC amplitudes and frequencies recorded from WT, presynaptic and postsynaptic BDNF KO neurons treated with DMSO or RA. ***, *P*<0.001; two-way ANOVA followed by Tukey’s test. Amp: F(2,199) = 1.09, *P*>0.3; freq: F(2,199) = 6.24, *P*<0.005. (**C**) Quantification of mEPSC amplitudes and frequencies recorded from WT, presynaptic and postsynaptic BDNF KO neurons treated with DMSO or CNQX (36 hours). **, *P*<0.01; ***, *P*<0.001; two-way ANOVA followed by Tukey’s test. Amp: F(2,195) = 1.77, *P*>0.15; freq: F(2,195) = 5.21, *P*<0.01. (**D**) Example traces of mEPSCs recorded from hippocampal pyramidal neurons in organotypic slices from WT (uninfected), presynaptic TrkB KO (Cre expression in CA3) and postsynaptic TrkB KO (Cre expression in CA1) groups treated with DMSO or RA (10 μM, 4 hr). Scale bars, 10 pA, 0.5 sec. (**E**) Quantification of mEPSC amplitudes and frequencies recorded from WT, presynaptic and postsynaptic TrkB KO neurons treated with DMSO or RA. **, *P*<0.01; ***, *P*<0.001; two-way ANOVA followed by Tukey’s test. Amp: F(2,164) = 0.14, *P*>0.8; freq: F(2,164) = 12.20, *P*<0.0001. (**F**) Quantification of mEPSC amplitudes and frequencies recorded from WT, presynaptic and postsynaptic TrkB KO neurons treated with DMSO or CNQX (36 hours). **, *P*<0.01; ***, *P*<0.001; two-way ANOVA followed by Tukey’s test. Amp: F(2,180) = 1.72, *P*>0.15; freq: F(2,180) = 3.62, *P*<0.05. n/N represent number of neurons/number of independent experiments. All graphs represent mean ± SEM.

The article has been corrected accordingly.

