# Supplementary material for: Retinoic acid-gated BDNF synthesis in neuronal dendrites drives presynaptic homeostatic plasticity
Source: eLife. 2022 Dec 14;11:e79863. doi: 10.7554/eLife.79863 (PMC9797192; doi:10.7554/eLife.79863)
Supplement: Figure 2—source data 2. — Figure 2A Actin and FigureS2A Actin: Immunoblots depicting actin expression profile in cultured hippocampal slices. Figure 2C Coommassie: Coomassie brilliant blue-stained SDS-polyacrylamide gel showing the expression of purified recombinant proteins. Figure 2D GluR1: Representative image for semi-quantitative RT-PCR of GluA1 in in vitro selection assay. Figure 2D BDNF Exon 1: Representative image for semi-quantitative RT-PCR of Bdnf exon 1 in in vitro selection assay. Figure 2D BDNF Exon 2: Representative image for semi-quantitative RT-PCR of Bdnf exon 2 in in vitro selection assay. Figure 2D BDNF Exon 6: Representative image for semi-quantitative RT-PCR of Bdnf exon 6 in in vitro selection assay. Figure 2D CamKII PSD95: Representative image for semi-quantitative RT-PCR of Psd95 and Camkii in in vitro selection assay. Figure 2D EF1a: Representative image for semi-quantitative RT-PCR of Ef1a in in vitro selection assay. Figure 2E ProBDNF: Immunoblot showing proBDNF synthesis in synaptoneurosomal fraction following retinoic acid (RA) treatment. Figure 2E Actin: Immunoblot showing actin levels in synaptoneurosomal fraction following RA treatment. [file elife-79863-fig2-data2.zip › Figure 2 cropped and labelled gel images.pptx]

## Slide 1
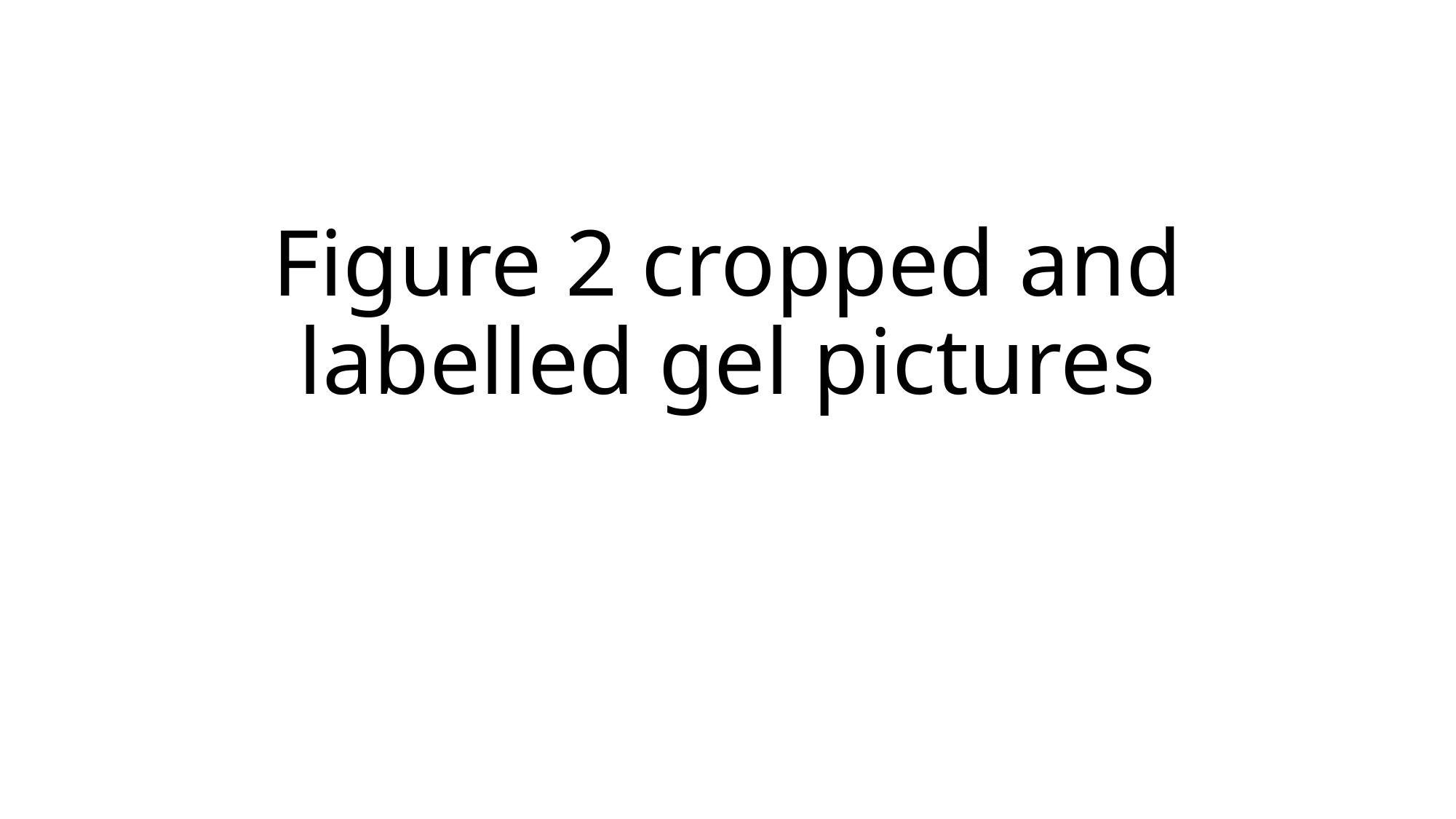

# Figure 2 cropped and labelled gel pictures

## Slide 2
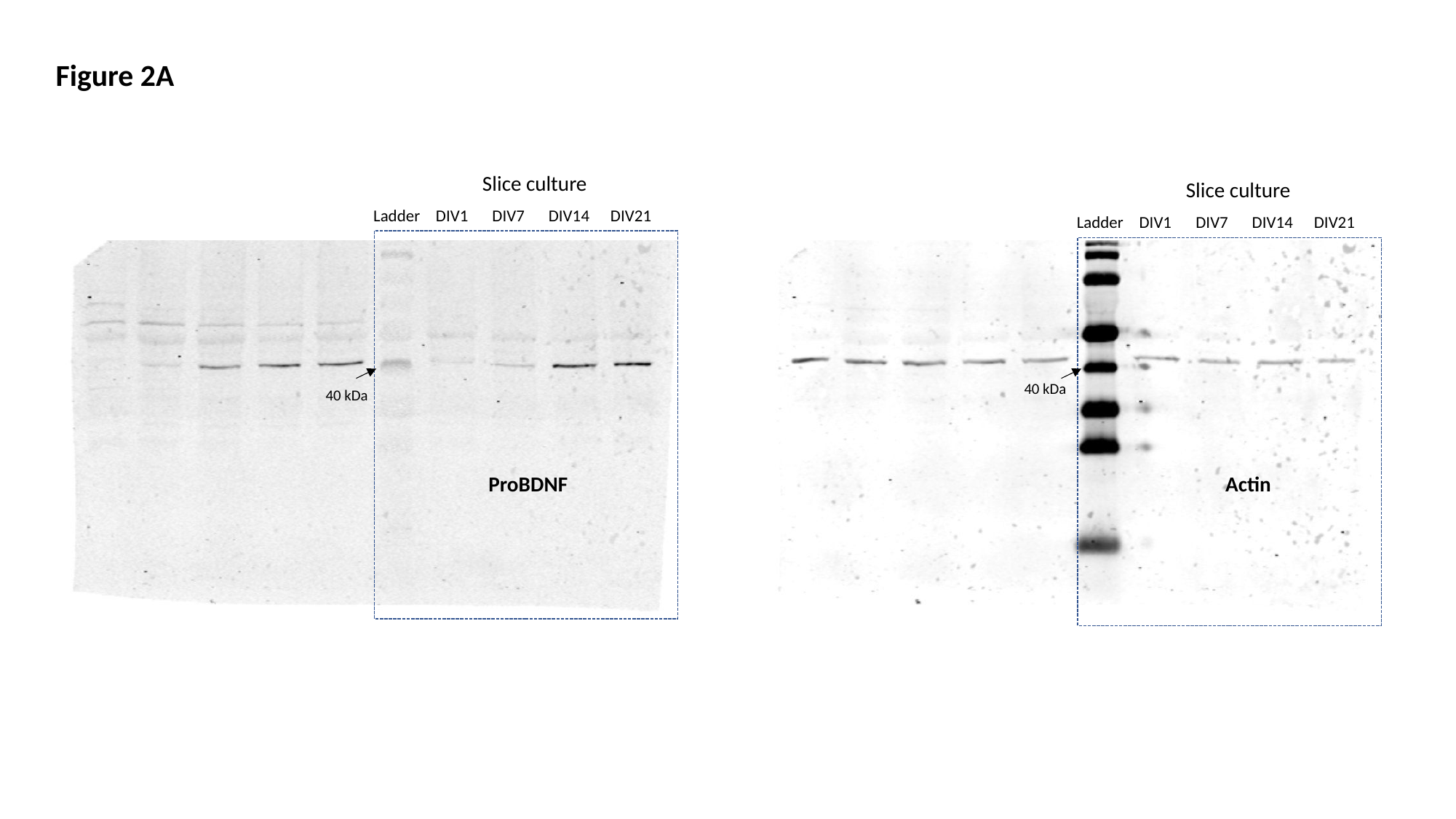

Figure 2A
Slice culture
Slice culture
Ladder
DIV1
DIV7
DIV14
DIV21
Ladder
DIV1
DIV7
DIV14
DIV21
40 kDa
40 kDa
ProBDNF
Actin

## Slide 3
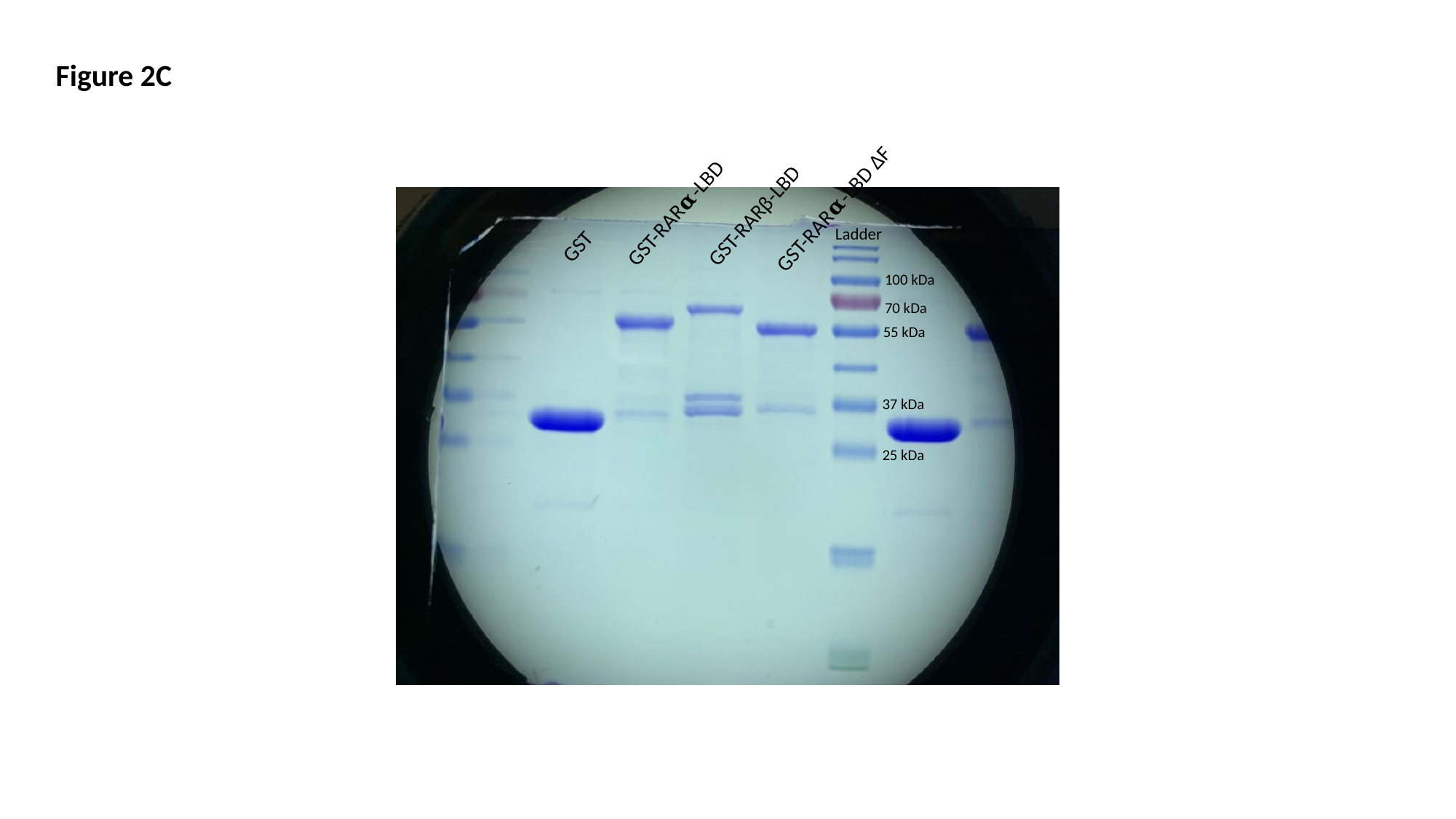

Figure 2C
GST-RARβ-LBD
GST-RAR𝛂-LBD
GST-RAR𝛂-LBD ΔF
GST
Ladder
100 kDa
70 kDa
55 kDa
37 kDa
25 kDa

## Slide 4
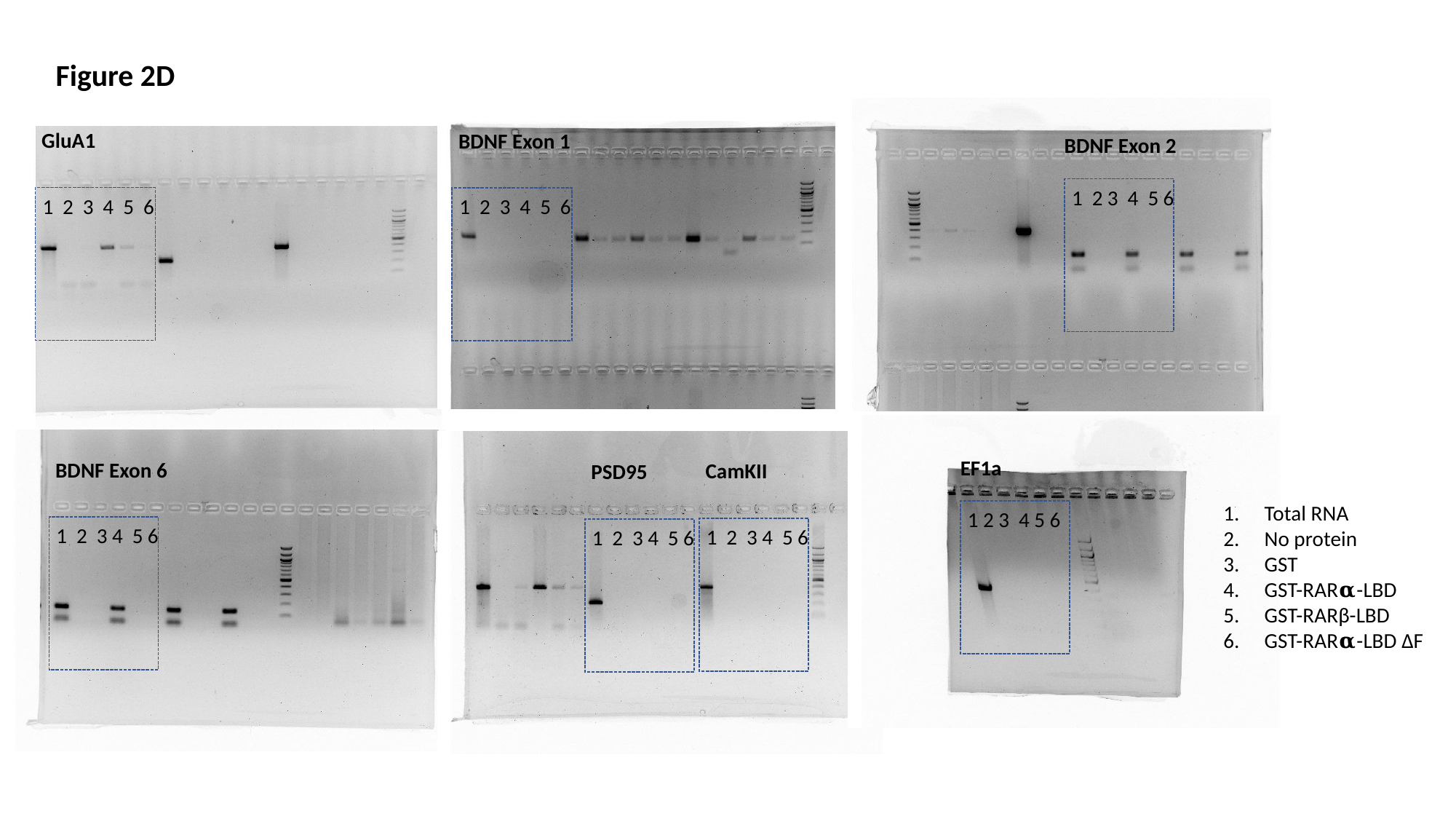

Figure 2D
GluA1
BDNF Exon 1
BDNF Exon 2
1 2 3 4 5 6
1 2 3 4 5 6
1 2 3 4 5 6
EF1a
BDNF Exon 6
CamKII
PSD95
Total RNA
No protein
GST
GST-RAR𝛂-LBD
GST-RARβ-LBD
GST-RAR𝛂-LBD ΔF
1 2 3 4 5 6
1 2 3 4 5 6
1 2 3 4 5 6
1 2 3 4 5 6

## Slide 5
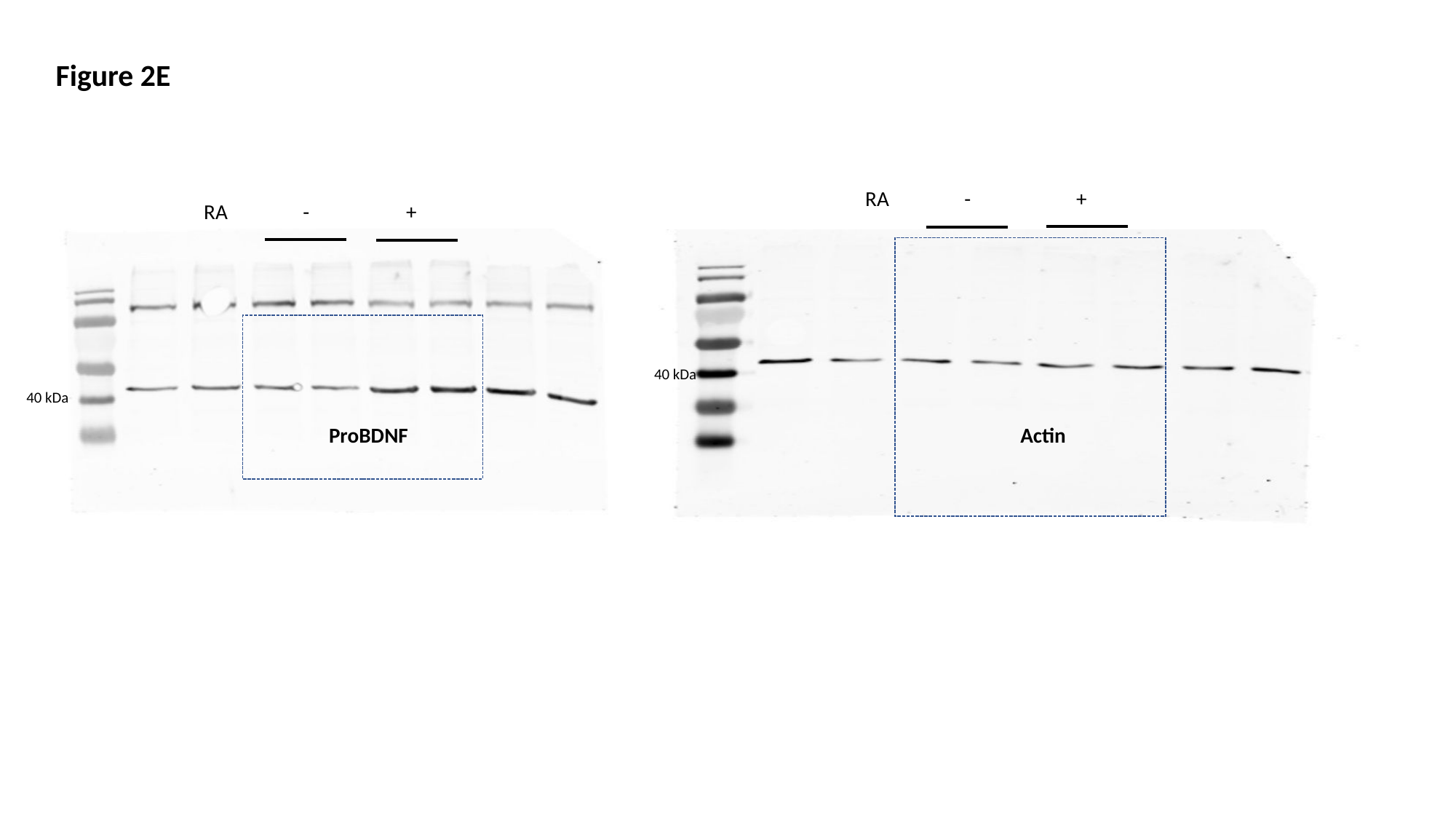

Figure 2E
+
-
RA
+
-
RA
40 kDa
40 kDa
ProBDNF
Actin
