# Supplementary figures and images for: Retinoic acid-gated BDNF synthesis in neuronal dendrites drives presynaptic homeostatic plasticity
Source: eLife. 2022 Dec 14;11:e79863. doi: 10.7554/eLife.79863 (PMC9797192; doi:10.7554/eLife.79863)

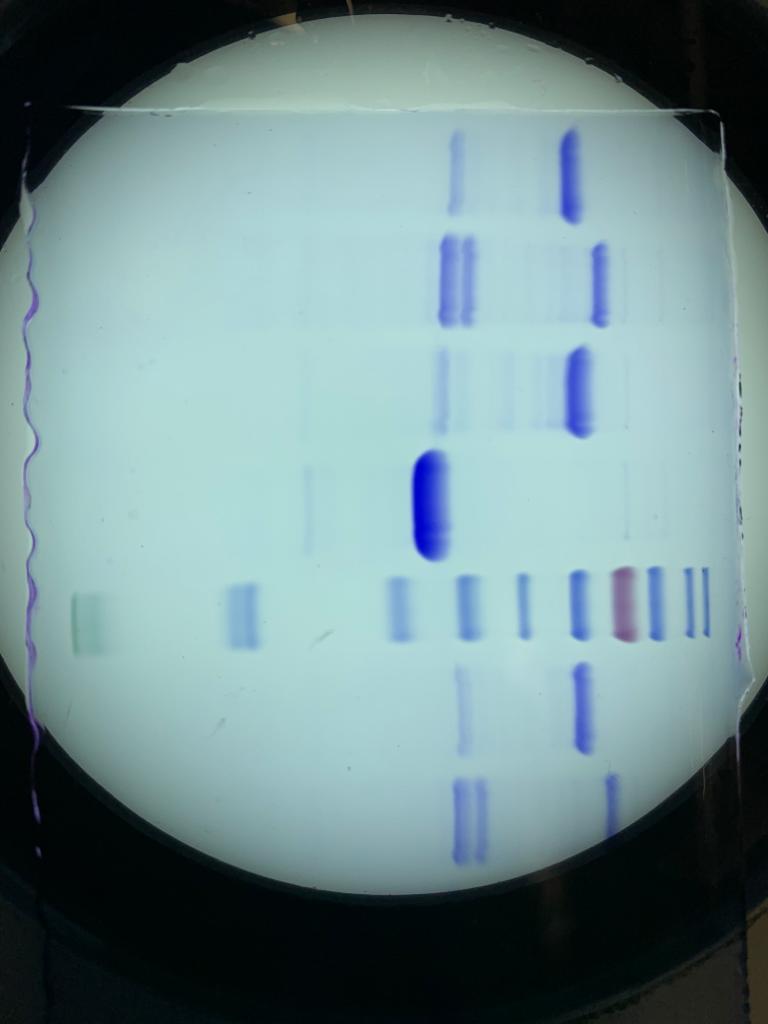

Supplement: Figure 2—source data 2. — Figure 2A Actin and FigureS2A Actin: Immunoblots depicting actin expression profile in cultured hippocampal slices. Figure 2C Coommassie: Coomassie brilliant blue-stained SDS-polyacrylamide gel showing the expression of purified recombinant proteins. Figure 2D GluR1: Representative image for semi-quantitative RT-PCR of GluA1 in in vitro selection assay. Figure 2D BDNF Exon 1: Representative image for semi-quantitative RT-PCR of Bdnf exon 1 in in vitro selection assay. Figure 2D BDNF Exon 2: Representative image for semi-quantitative RT-PCR of Bdnf exon 2 in in vitro selection assay. Figure 2D BDNF Exon 6: Representative image for semi-quantitative RT-PCR of Bdnf exon 6 in in vitro selection assay. Figure 2D CamKII PSD95: Representative image for semi-quantitative RT-PCR of Psd95 and Camkii in in vitro selection assay. Figure 2D EF1a: Representative image for semi-quantitative RT-PCR of Ef1a in in vitro selection assay. Figure 2E ProBDNF: Immunoblot showing proBDNF synthesis in synaptoneurosomal fraction following retinoic acid (RA) treatment. Figure 2E Actin: Immunoblot showing actin levels in synaptoneurosomal fraction following RA treatment. [file elife-79863-fig2-data2.zip › Figure 2C Coommassie.JPG]

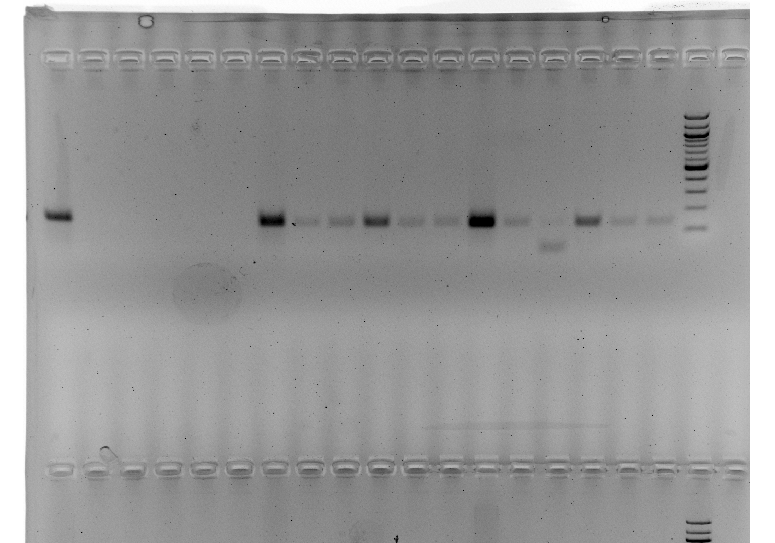

Supplement: Figure 2—source data 2. — Figure 2A Actin and FigureS2A Actin: Immunoblots depicting actin expression profile in cultured hippocampal slices. Figure 2C Coommassie: Coomassie brilliant blue-stained SDS-polyacrylamide gel showing the expression of purified recombinant proteins. Figure 2D GluR1: Representative image for semi-quantitative RT-PCR of GluA1 in in vitro selection assay. Figure 2D BDNF Exon 1: Representative image for semi-quantitative RT-PCR of Bdnf exon 1 in in vitro selection assay. Figure 2D BDNF Exon 2: Representative image for semi-quantitative RT-PCR of Bdnf exon 2 in in vitro selection assay. Figure 2D BDNF Exon 6: Representative image for semi-quantitative RT-PCR of Bdnf exon 6 in in vitro selection assay. Figure 2D CamKII PSD95: Representative image for semi-quantitative RT-PCR of Psd95 and Camkii in in vitro selection assay. Figure 2D EF1a: Representative image for semi-quantitative RT-PCR of Ef1a in in vitro selection assay. Figure 2E ProBDNF: Immunoblot showing proBDNF synthesis in synaptoneurosomal fraction following retinoic acid (RA) treatment. Figure 2E Actin: Immunoblot showing actin levels in synaptoneurosomal fraction following RA treatment. [file elife-79863-fig2-data2.zip › Figure 2D BDNF Exon 1.tif]

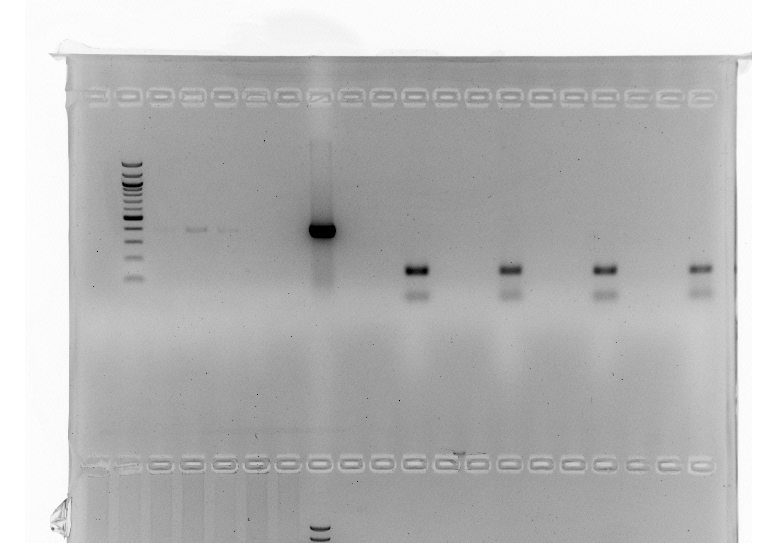

Supplement: Figure 2—source data 2. — Figure 2A Actin and FigureS2A Actin: Immunoblots depicting actin expression profile in cultured hippocampal slices. Figure 2C Coommassie: Coomassie brilliant blue-stained SDS-polyacrylamide gel showing the expression of purified recombinant proteins. Figure 2D GluR1: Representative image for semi-quantitative RT-PCR of GluA1 in in vitro selection assay. Figure 2D BDNF Exon 1: Representative image for semi-quantitative RT-PCR of Bdnf exon 1 in in vitro selection assay. Figure 2D BDNF Exon 2: Representative image for semi-quantitative RT-PCR of Bdnf exon 2 in in vitro selection assay. Figure 2D BDNF Exon 6: Representative image for semi-quantitative RT-PCR of Bdnf exon 6 in in vitro selection assay. Figure 2D CamKII PSD95: Representative image for semi-quantitative RT-PCR of Psd95 and Camkii in in vitro selection assay. Figure 2D EF1a: Representative image for semi-quantitative RT-PCR of Ef1a in in vitro selection assay. Figure 2E ProBDNF: Immunoblot showing proBDNF synthesis in synaptoneurosomal fraction following retinoic acid (RA) treatment. Figure 2E Actin: Immunoblot showing actin levels in synaptoneurosomal fraction following RA treatment. [file elife-79863-fig2-data2.zip › Figure 2D BDNF Exon 2.tif]

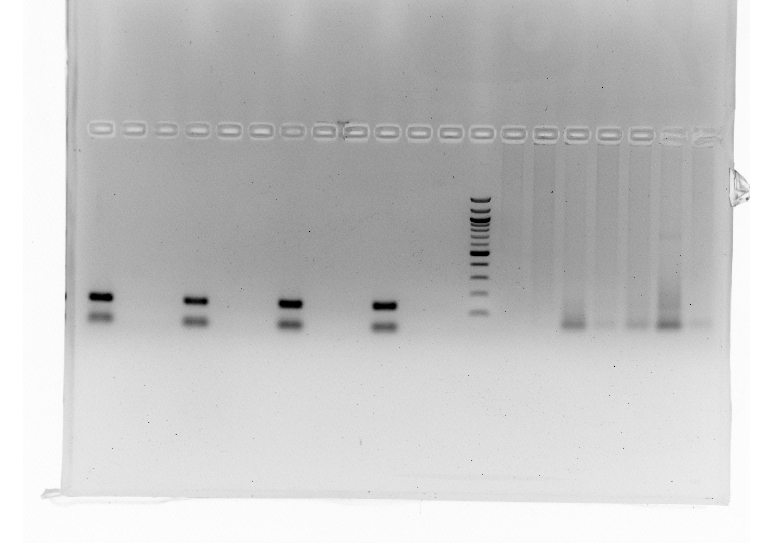

Supplement: Figure 2—source data 2. — Figure 2A Actin and FigureS2A Actin: Immunoblots depicting actin expression profile in cultured hippocampal slices. Figure 2C Coommassie: Coomassie brilliant blue-stained SDS-polyacrylamide gel showing the expression of purified recombinant proteins. Figure 2D GluR1: Representative image for semi-quantitative RT-PCR of GluA1 in in vitro selection assay. Figure 2D BDNF Exon 1: Representative image for semi-quantitative RT-PCR of Bdnf exon 1 in in vitro selection assay. Figure 2D BDNF Exon 2: Representative image for semi-quantitative RT-PCR of Bdnf exon 2 in in vitro selection assay. Figure 2D BDNF Exon 6: Representative image for semi-quantitative RT-PCR of Bdnf exon 6 in in vitro selection assay. Figure 2D CamKII PSD95: Representative image for semi-quantitative RT-PCR of Psd95 and Camkii in in vitro selection assay. Figure 2D EF1a: Representative image for semi-quantitative RT-PCR of Ef1a in in vitro selection assay. Figure 2E ProBDNF: Immunoblot showing proBDNF synthesis in synaptoneurosomal fraction following retinoic acid (RA) treatment. Figure 2E Actin: Immunoblot showing actin levels in synaptoneurosomal fraction following RA treatment. [file elife-79863-fig2-data2.zip › Figure 2D BDNF Exon 6.tif]

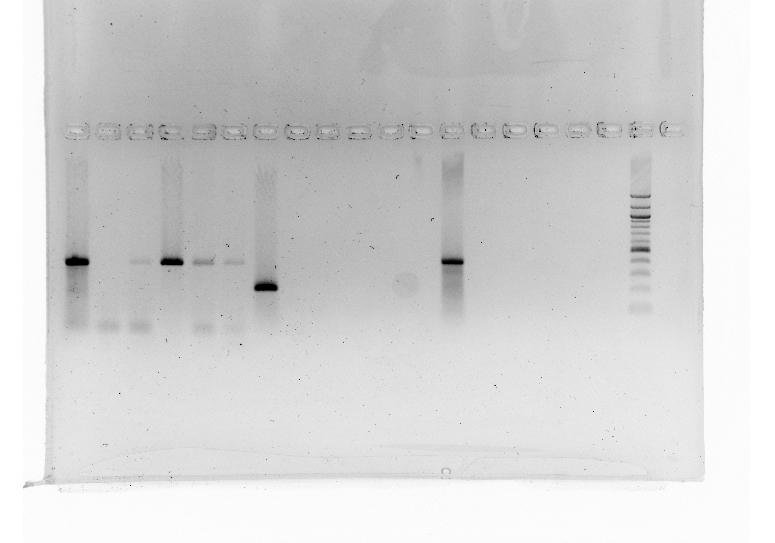

Supplement: Figure 2—source data 2. — Figure 2A Actin and FigureS2A Actin: Immunoblots depicting actin expression profile in cultured hippocampal slices. Figure 2C Coommassie: Coomassie brilliant blue-stained SDS-polyacrylamide gel showing the expression of purified recombinant proteins. Figure 2D GluR1: Representative image for semi-quantitative RT-PCR of GluA1 in in vitro selection assay. Figure 2D BDNF Exon 1: Representative image for semi-quantitative RT-PCR of Bdnf exon 1 in in vitro selection assay. Figure 2D BDNF Exon 2: Representative image for semi-quantitative RT-PCR of Bdnf exon 2 in in vitro selection assay. Figure 2D BDNF Exon 6: Representative image for semi-quantitative RT-PCR of Bdnf exon 6 in in vitro selection assay. Figure 2D CamKII PSD95: Representative image for semi-quantitative RT-PCR of Psd95 and Camkii in in vitro selection assay. Figure 2D EF1a: Representative image for semi-quantitative RT-PCR of Ef1a in in vitro selection assay. Figure 2E ProBDNF: Immunoblot showing proBDNF synthesis in synaptoneurosomal fraction following retinoic acid (RA) treatment. Figure 2E Actin: Immunoblot showing actin levels in synaptoneurosomal fraction following RA treatment. [file elife-79863-fig2-data2.zip › Figure 2D CamKII PSD95.tif]

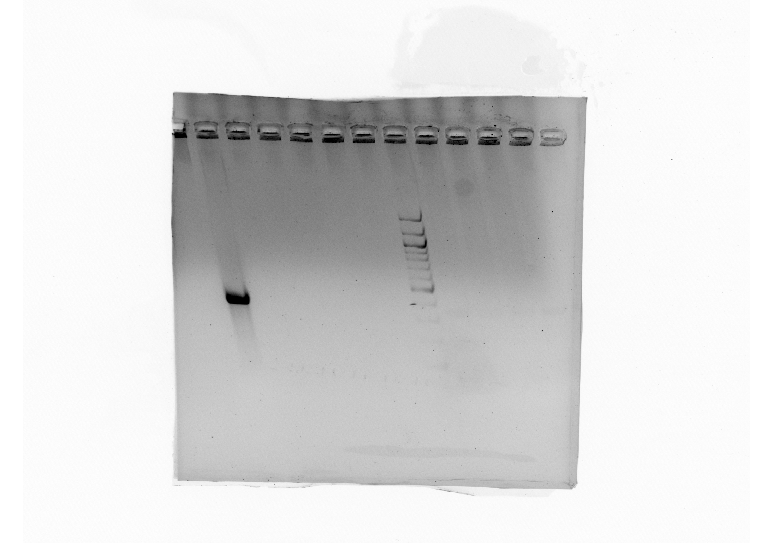

Supplement: Figure 2—source data 2. — Figure 2A Actin and FigureS2A Actin: Immunoblots depicting actin expression profile in cultured hippocampal slices. Figure 2C Coommassie: Coomassie brilliant blue-stained SDS-polyacrylamide gel showing the expression of purified recombinant proteins. Figure 2D GluR1: Representative image for semi-quantitative RT-PCR of GluA1 in in vitro selection assay. Figure 2D BDNF Exon 1: Representative image for semi-quantitative RT-PCR of Bdnf exon 1 in in vitro selection assay. Figure 2D BDNF Exon 2: Representative image for semi-quantitative RT-PCR of Bdnf exon 2 in in vitro selection assay. Figure 2D BDNF Exon 6: Representative image for semi-quantitative RT-PCR of Bdnf exon 6 in in vitro selection assay. Figure 2D CamKII PSD95: Representative image for semi-quantitative RT-PCR of Psd95 and Camkii in in vitro selection assay. Figure 2D EF1a: Representative image for semi-quantitative RT-PCR of Ef1a in in vitro selection assay. Figure 2E ProBDNF: Immunoblot showing proBDNF synthesis in synaptoneurosomal fraction following retinoic acid (RA) treatment. Figure 2E Actin: Immunoblot showing actin levels in synaptoneurosomal fraction following RA treatment. [file elife-79863-fig2-data2.zip › Figure 2D EF1a.tif]

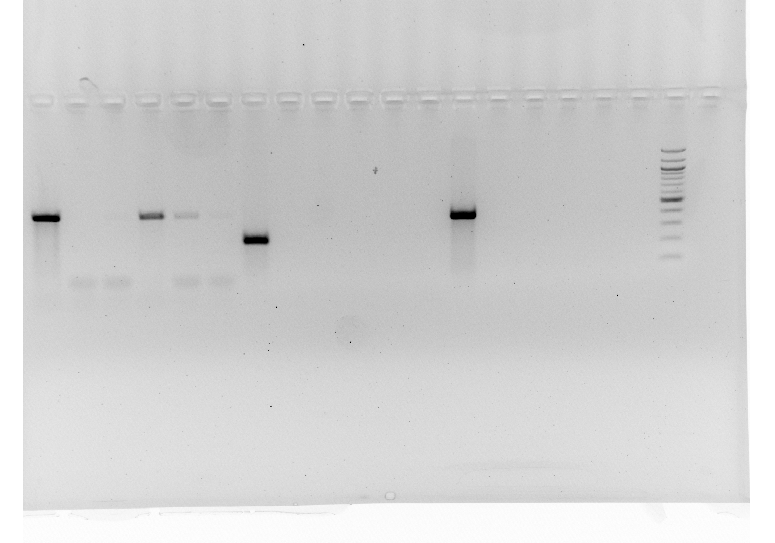

Supplement: Figure 2—source data 2. — Figure 2A Actin and FigureS2A Actin: Immunoblots depicting actin expression profile in cultured hippocampal slices. Figure 2C Coommassie: Coomassie brilliant blue-stained SDS-polyacrylamide gel showing the expression of purified recombinant proteins. Figure 2D GluR1: Representative image for semi-quantitative RT-PCR of GluA1 in in vitro selection assay. Figure 2D BDNF Exon 1: Representative image for semi-quantitative RT-PCR of Bdnf exon 1 in in vitro selection assay. Figure 2D BDNF Exon 2: Representative image for semi-quantitative RT-PCR of Bdnf exon 2 in in vitro selection assay. Figure 2D BDNF Exon 6: Representative image for semi-quantitative RT-PCR of Bdnf exon 6 in in vitro selection assay. Figure 2D CamKII PSD95: Representative image for semi-quantitative RT-PCR of Psd95 and Camkii in in vitro selection assay. Figure 2D EF1a: Representative image for semi-quantitative RT-PCR of Ef1a in in vitro selection assay. Figure 2E ProBDNF: Immunoblot showing proBDNF synthesis in synaptoneurosomal fraction following retinoic acid (RA) treatment. Figure 2E Actin: Immunoblot showing actin levels in synaptoneurosomal fraction following RA treatment. [file elife-79863-fig2-data2.zip › Figure 2D GluR1.tif]
