# Supplementary material for: Retinoic acid-gated BDNF synthesis in neuronal dendrites drives presynaptic homeostatic plasticity
Source: eLife. 2022 Dec 14;11:e79863. doi: 10.7554/eLife.79863 (PMC9797192; doi:10.7554/eLife.79863)
Supplement: Figure 2—figure supplement 1—source data 2. — Figure 2A Actin and Figure S2A Actin: Immunoblots depicting actin expression profile in whole hippocampi collected from mouse pups. Figure 2: GST-immunoblot showing the expression levels of purified recombinant proteins. FigureS2D Histone H3: Immunoblot to confirm histone H3 was selectively absent from the hippocampal synaptoneurosome fraction relative to the whole-cell lysate. Figure 2 PSD95: Immunoblot to confirm PSD95 was enriched in the hippocampal synaptoneurosome fraction relative to the whole-cell lysate. FigureS2D Actin: Immunoblot depicting actin levels in hippocampal synaptoneurosome fraction and whole-cell lysate. FigureS2E GluA1 and actin: Immunoblot showing GluA1 synthesis in synaptoneurosomal fraction following retinoic acid (RA) treatment. Actin is shown as a loading control. FigureS2F PSD95 and actin: Immunoblot showing no PSD95 synthesis in synaptoneurosomal fraction following RA treatment. Actin is shown as loading control. [file elife-79863-fig2-figsupp1-data2.zip › Figure 2 - figure supplement 1 - cropped and labelled images.pptx]

## Slide 1
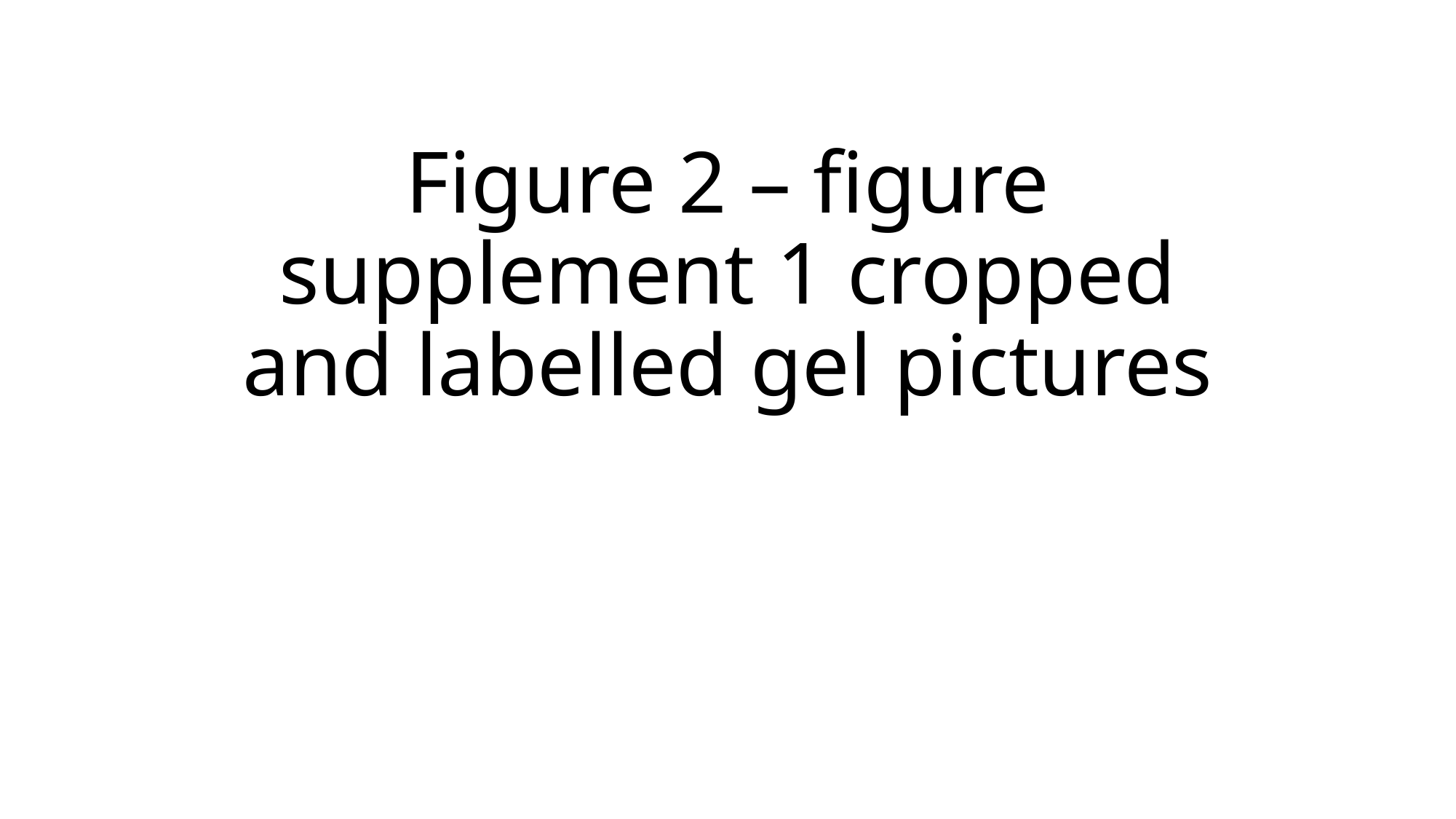

# Figure 2 – figure supplement 1 cropped and labelled gel pictures

## Slide 2
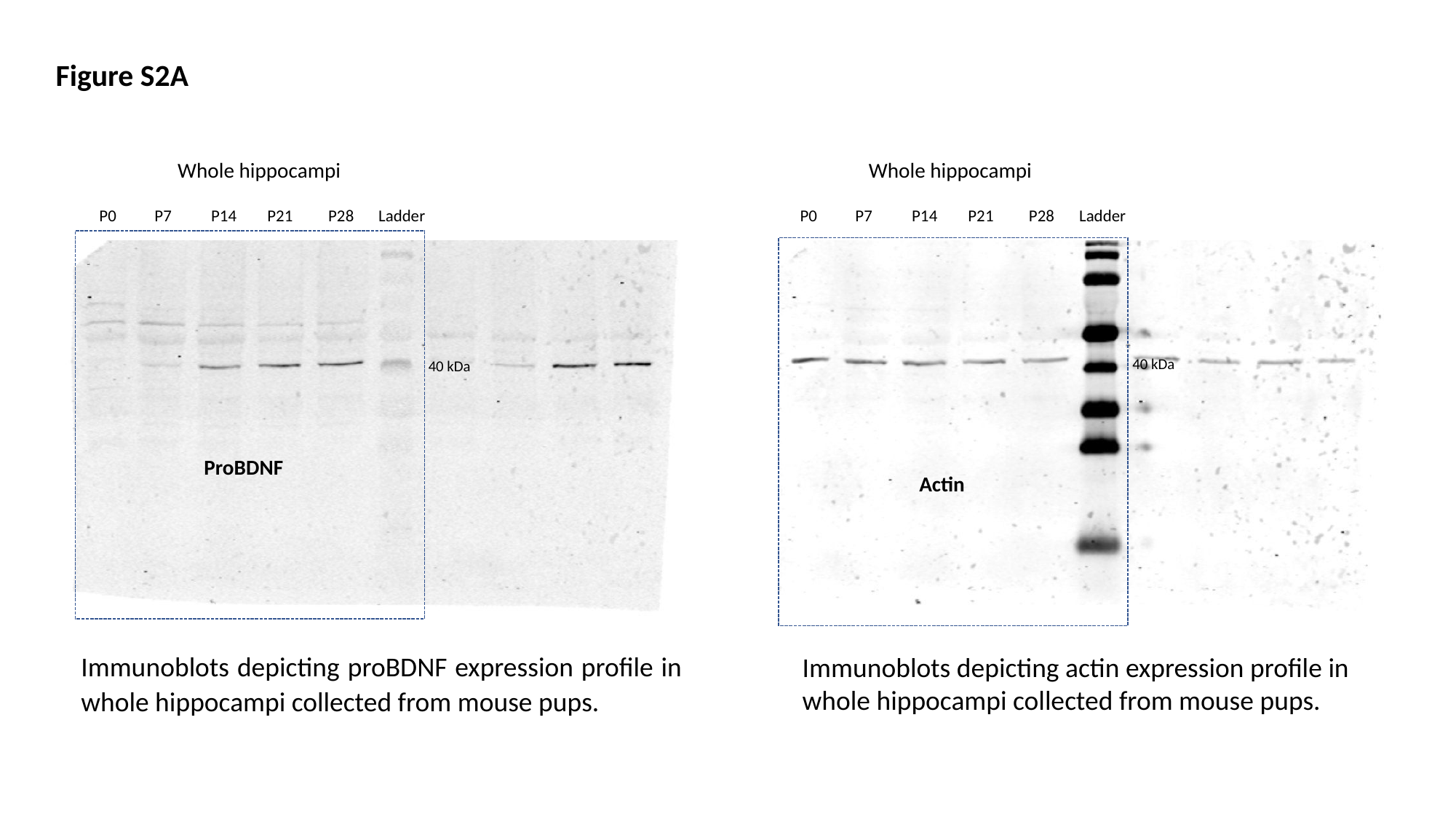

Figure S2A
Whole hippocampi
Whole hippocampi
P0
P7
P14
P21
P28
Ladder
P0
P7
P14
P21
P28
Ladder
40 kDa
40 kDa
ProBDNF
Actin
Immunoblots depicting proBDNF expression profile in whole hippocampi collected from mouse pups.
Immunoblots depicting actin expression profile in whole hippocampi collected from mouse pups.

## Slide 3
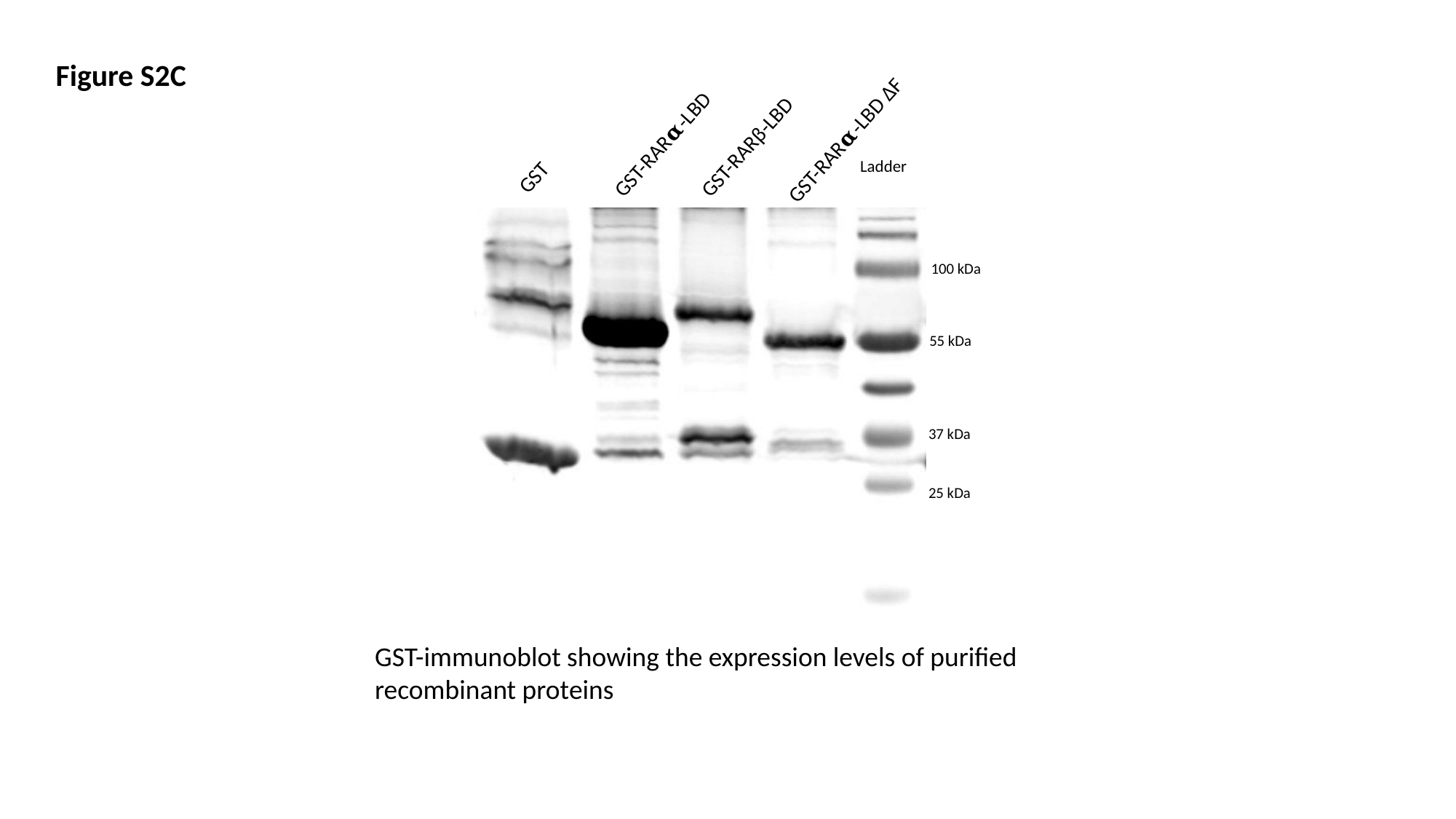

Figure S2C
GST-RARβ-LBD
GST-RAR𝛂-LBD
GST-RAR𝛂-LBD ΔF
GST
Ladder
100 kDa
55 kDa
37 kDa
25 kDa
GST-immunoblot showing the expression levels of purified recombinant proteins

## Slide 4
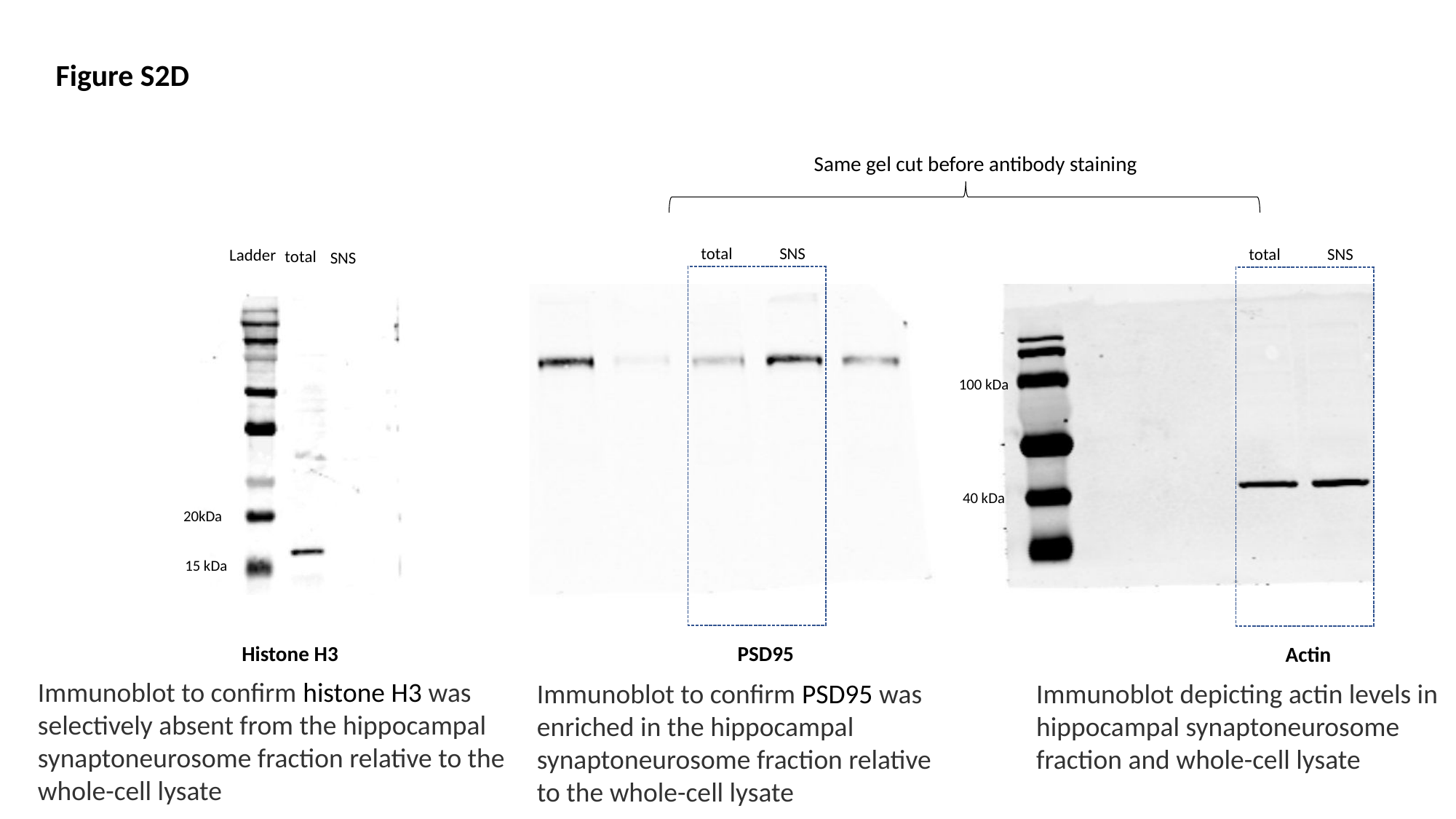

Figure S2D
Same gel cut before antibody staining
total
SNS
total
SNS
Ladder
total
SNS
100 kDa
40 kDa
20kDa
15 kDa
Histone H3
PSD95
Actin
Immunoblot to confirm histone H3 was selectively absent from the hippocampal synaptoneurosome fraction relative to the whole-cell lysate
Immunoblot depicting actin levels in hippocampal synaptoneurosome fraction and whole-cell lysate
Immunoblot to confirm PSD95 was enriched in the hippocampal synaptoneurosome fraction relative to the whole-cell lysate

## Slide 5
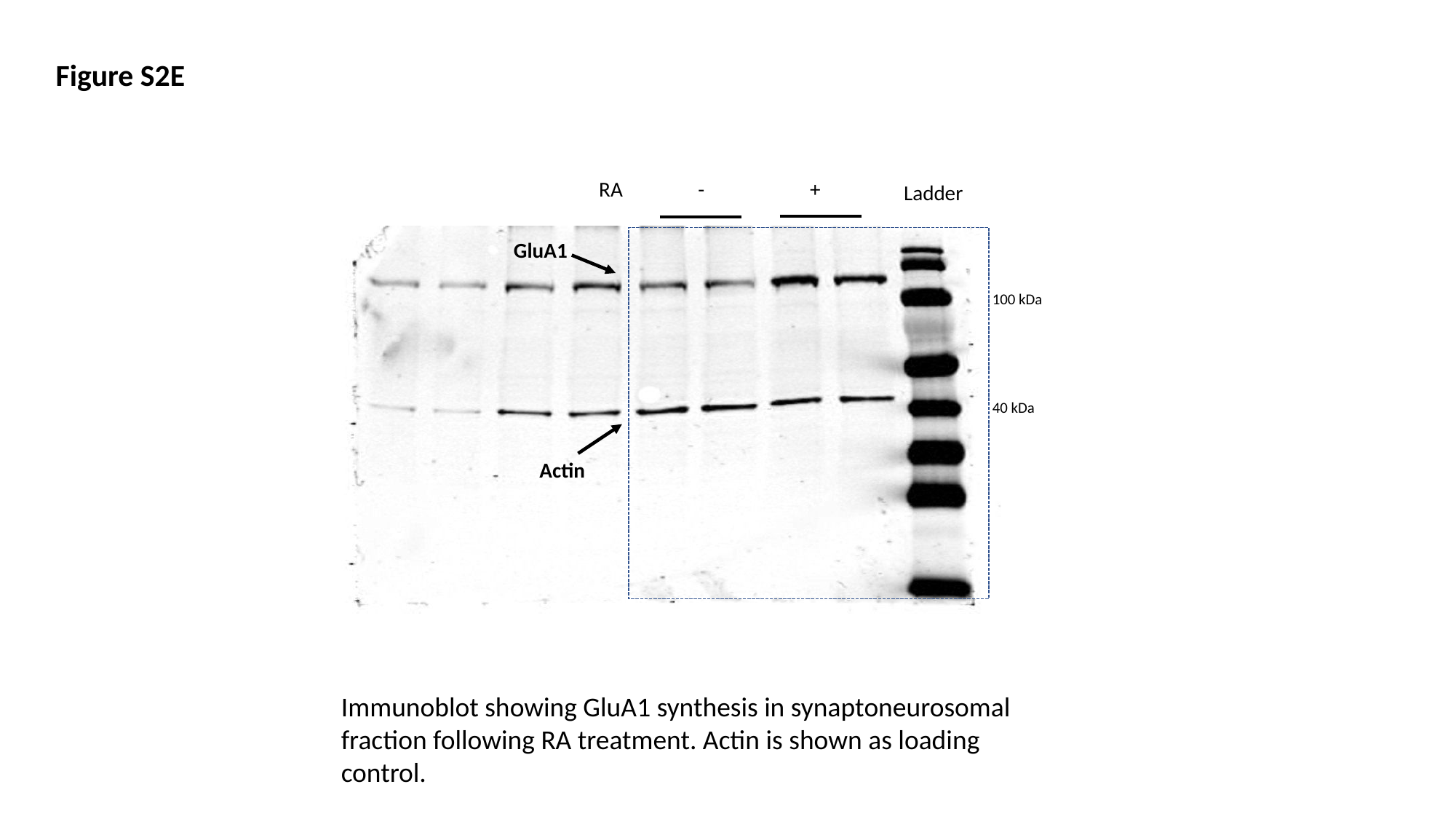

Figure S2E
+
-
RA
Ladder
GluA1
100 kDa
40 kDa
Actin
Immunoblot showing GluA1 synthesis in synaptoneurosomal fraction following RA treatment. Actin is shown as loading control.

## Slide 6
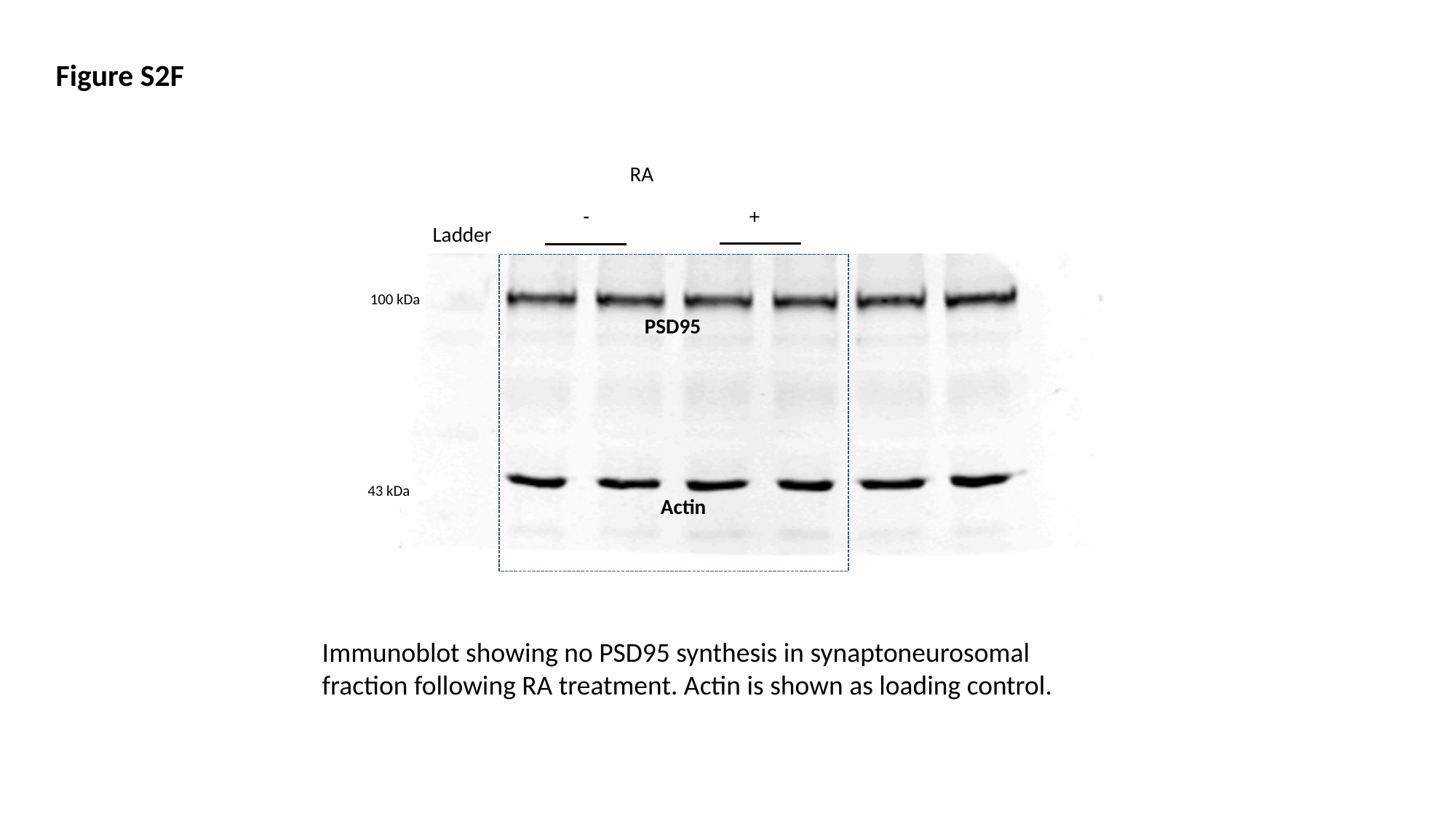

Figure S2F
RA
+
-
Ladder
100 kDa
PSD95
43 kDa
Actin
Immunoblot showing no PSD95 synthesis in synaptoneurosomal fraction following RA treatment. Actin is shown as loading control.
